# Extracts from the Report of the Committee Employed to Visit Houses and Hospitals for the Confinement of Insane Persons, with Remarks

**Published:** 1814-08

**Authors:** 


*For the Medical and Physical Journal.*

### *Extracts from the Report of the Committee employed to visit Houses and Hospitals for the Confinement of Insane Persons, with Remarks; by* PHILANTHROPOS.

IT was announced some time ago in the Medical Journal, that a meeting had been holden for the purpose of establishing an Asylum for the Care and Cure of the Insane in London. The necessity of such a measure, at that time, did not appear very obvious to those persons who were not acquainted with the nature and regulations of the existing establishments, either public or private, for the reception of insane persons, or of the great number of individuals requiring confinement. A committee was appointed to investigate the subject, and they have made a report which every philanthropist would wish to be made as public as possible, because it displays a system of negligence and cruelty which must excite universal horror, and consequently cause its immediate abolition. To enable the profession to form a just opinion of the case, as well as to appreciate the meritorious efforts of the committee, a few extracts from their report will for the present suffice. They will attest the labours of the committee, and tend to explain the probable benefit which will result from their exertions, whilst there can be no doubt that the parties who are culpable will be sufficiently aware of the effect of public opinion, very speedily to to adopt more humane and effectual measures for the relief of the unfortunate sufferers entrusted to their care, than has hitherto been the case.

From the report of the committee, it appears that the usual modes of confining persons afflicted with insanity, are four:

1. At their own homes, or in the houses of their friends, attended by a keeper.

2. In work-houses, in which the patients are principally parish paupers.

3. In private receptacles.

4. In public hospitals purposely elected.

Of the first class, the committee, from the difficulties of obtaining information, forbore to make inquiry. Of the second class, they state that the patients "are generally kept in gloomy and comfortless confinement." But they do not point out any very important abuses.

Neither do they seem to have met with much cause of complaint in their investigation into the third class, or those confined in private houses.

But that part of their report which includes the public hospitals, deserves the most profound attention. "The two great public hospitals, St. Luke's and Bethlem, and the ward for the insane at Guy's Hospital, have been visited by the committee. The ward of Guy's Hospital, which is calculated to receive twenty female patients, termed incurables, is a separate building from the general hospital; and is, by being adapted to afford the keeper a complete and constant inspection of every part, by superior modes of ventilation, and in all other respects more suitably constructed than any erection for the insane within the bills of mortality. The management also appeared much better to the committee than what they observed in any other establishment. St. Luke's Hospital is by no means equal in accommodation to the ward at Guy's. Notwithstanding the superior activity and diligence of the present master, Mr. Thomas Dunston, and the great attention paid to cleanliness and ventilation, which the committee cannot praise too highly, the defects of the building must preclude him from realizing the judicious views which it appears, by his evidence before a committee of the House of Commons, in 1807, he entertains of the disease. 'Much,' says he, 'is certainly to be done by management; but it is impossible, on this head, to lay down a general rule; each effort must be adapted to the peculiar indisposition; it is necessary to check some, to encourage others, and to animate all with a hope of recovery.' This edifice has most of the radical evils of inadequate construction. The day-rooms are too small for the winter season, and the windows of most of the cells are unglazed; only one airing ground is allotted to each sex; and the galleries, all communicating with each other, preclude proper classification."

The committee encountered great difficulties in obtaining a view of the interior of Bethlem Hospital; at length, on Monday the 25th of April, they were introduced by one of the governors, being refused admission unless so accompanied; but he felt himself unable to attend them through; his feelings were quite overpowered. On the 2d of May the attempt was renewed, and the following is the description of what they witnessed. "One of the side rooms contained about ten patients, each chained by one arm to the wall; the chain allowing them merely to stand up by the bench or form fixed to the wall, or to sit down on it. The nakedness of each patient was covered by a blanket-gown only. The blanket-gown is a blanket formed something like a dressing gown, with nothing to fasten it with in front; this constitutes the whole covering; the feet even were naked. One female in this side room, thus chained, was an object remarkably striking; she mentioned her maiden and married names, and stated that she had been a teacher of languages. The keepers described her as a very accomplished lady, mistress of many languages, and corroborated her account of herself. The committee can hardly imagine a human being in a more degraded and humiliating situation, than that in which they found this female, who held a coherent conversation with them, and was, of course, fully sensible of the mental and bodily condition of those wretched beings who, equally without clothing, were closely chained to the same wall with herself. Unaware of the necessities of nature, some of them, though they contained life, appeared totally inanimate and unconscious of existence. The few minutes which the committee passed with this lady, did not permit them to form a judgment of the degree of restraint to which she ought to be subject; but they unhesitatingly affirm, that her confinement with patients, in whom she was compelled to witness the most disgusting idiocy, and the most terrifying distraction of the human intellect, is injudicious and improper. She entreated to be allowed pencil and paper, for the purpose of amusing herself with drawing, which were given her by one of the committee.

"Many other unfortunate women were locked up in their cells, naked, and chained on straw, with only one blanket for a covering. One, who was in that state by way of punishment, the keeper described as the most dissatisfied patient tient in the house; she talked coherently, complained of the want of tea and sugar, and lamented that her friends, whom she stated to be respectable people, neither came to see her, nor supplied her with little necessary comforts. The patients generally complained much of being deprived of tea and sugar. On leaving the gallery, the committee inquired of them, whether the visit had been inconvenient or unpleasant; they all joined in saying no, but (which was sufficiently apparent) that the visit of a friend was always pleasant.

"In the men's wing, in the side room, six patients were chained close to the wall—five hand-cuffed, and one locked to the wall by the right arm, as well as by the right leg; he was very noisy. All were naked, except as to the blanket gown, or a small rug on the shoulders, and without shoes; one complained much of the coldness of his feet,—one of the committee felt them—they were very cold. The patients in this room, except the noisy one, and the poor lad with cold feet, who was lucid when the committee saw him, were dreadful idiots. Their nakedness, and their mode of confinement, gave this room the complete appearance of a dog-kennel.

"From the patients not being classed, some appear objects of resentment to the others. The committee saw a quiet civil man, a soldier, a native of Poland, brutally attacked by another soldier, who, we were informed by the keepers, always singled out the Pole as an object of resentment.

"Chains are universally substituted for the straight waistcoat; but in Guy's Hospital, a leather belt is used, with side straps to confine the arms, which in many instances is greatly superior.

"In the men's wing were about 75 or 76 patients, with two keepers and an assistant; and about the same number of patients on the women's side. The patients were in no way distinguished from each other as to disease, than as those who were not walking about, or chained in the side rooms, were lying stark naked upon straw, on their bedsteads, each in a separate cell, with a single blanket or rug, in which the patient usually lay huddled up as if impatient of cold, and generally chained to the bed-place, in the shape of a trough. About one fifth were in this state, or chained in the side rooms. In the private houses, the patients are universally made to rise, to wear clothes, to take exercise, and, from being confined in a waistcoat when necessary, are prevented injuring each other.

It appeared, that the wet patients, and all who were inclined clined to be a-bed, were allowed to do so, from being less troublesome in that state than when up and dressed.

The end window towards Fore-street was the chief source of entertainment to the patients; they seemed greatly to enjoy the sight of the people walking, and to derive great pleasure from the visits of the committee.

In one of the cells of the lower gallery, the committee saw William Norris. He stated himself to be 55 years of age, and that he had been confined about fourteen years; that in consequence of attempting to defend himself from what he conceived improper treatment of his keeper, he was fastened by a long chain, which, passing through a partition, enabled the keeper, by going into the next cell, to draw him close to the wall at pleasure; that, to prevent this, Norris muffled the chain with straw, so as to hinder its passing through the wall; that he afterwards was confined in the manner the committee *saw him*, viz. a stout iron ring was rivetted round his neck, from which a short chain passed to a ring, made to slide upwards or downwards on an upright massive iron bar, more than six feet high, inserted into the wall; round his body, a strong iron bar, about two inches wide, was rivetted; on each side the bar was a circular projection, which being fashioned to, and enclosing each of his arms, pinioned them close to his sides; this waist-bar was secured by two similar bars, which passing over his shoulders were rivetted to the waist-bar both before and behind; the iron ring round his neck was connected to the bars on his shoulders by a double link; from each of these bars another short chain passed to the ring on the upright bar. We were informed he was enabled to raise himself, so as to stand against the wall, on the pillow of his bed, in the trough-bed in which he lay; but it is impossible for him to advance from the wall in which the iron bar is soldered, on account of the shortness of his chains, which were only twelve inches long. It is conceived equally out of his power to repose in any other position than on his back, the projections which on each side of the waist-bar enclosed his arms, rendering it impossible for him to lay on his side, even if the length of the chains from his neck and shoulders would permit it. His right leg was chained to the trough, in which he had remained thus encaged and chained more than twelve years. To prove the unnecessary restraints inflicted on this unfortunate man, he informed the committee, that he had for some years been able to withdraw his arms from the manacles which encompassed them. He then withdrew one of them; and, observing an expression of surprise, he said, that when his arms were withdrawn, he was compelled to rest them on the edges of of the circular projections, which was more painful than keeping them within. His position, we were informed, was mostly lying down, and that, as it was inconvenient to raise himself and stand upright, he very seldom did so; that he read a great deal, books of all kinds, history, lives, or any thing that the keepers could get him, the newspaper every day, and conversed perfectly coherent on the passing topics and events of the war, in which he felt particular interest. On each day that the committee saw him he discoursed coolly, and gave rational and deliberate answers to the different questions put to him. The whole of this statement relative to W. Norris was confirmed by the keepers."

On a subsequent visit they observed that the whole of the irons had been removed from Norris's body, and that the length of the chain from his neck, which was only 12 inches, had been doubled.

"In the public hospitals, it is customary to lock up the patients in their cells at dusk, this in winter is soon after four o'clock; and the cells are not opened until seven o'clock the next morning. The coldness of the season sends the patient into his bed, however he may incline to remain awake; to him, who is darkness and utter confusion within, this is no privation of comfort, no infliction of sorrow; but, surely, fifteen hours of dreary solitary confinement, in a dark cell, must tend to retard the progress of the convalescent, and to deepen the gloom of the mind, shattered by sorrow.

"If the committee have been pained by the remarkable contrast in management between one of our great public hospitals for the insane, and the larger private houses generally, they have been as forcibly impressed by contrasting the practice of even such houses with the general economy of the '*Friend's Retreat*,' near York; where neither chains nor corporeal punishment are tolerated on any pretext; where the conveniencies provided, both within doors and without, are suitable to patients in any station of life, and every appearance is avoided that can afflict the mind by painful recollection; and where regulation and controul are governed by the experienced efficacy of this important principle, that whatever tends to promote the happiness of the patient, increases his desire to restrain himself."

It is needless to make further extracts from this most important report. The committee have completely ascertained many gross abuses, have proved the whole system of management in one of our largest hospitals to be radically bad, and therefore have the strongest grounds for recommending a new asylum, to be conducted after the simple and mild, but but successful, plan of the "Friend's Retreat," at York; an institution which is likely to become instrumental in reforming the erroneous system of many others that have been longer established, but whose managers have not been influenced by humane and enlightened principles.

Before concluding this paper it may be proper to remark, that the committee have allowed some uncandid observations on physicians to escape them. If the college of physicians have illiberally secured to their own *fellows* the privileges and emoluments derived from visiting and licensing receptacles for lunatics, if they have on some occasions exercised their power in an arbitrary manner, or have not always evinced that sagacity and discretion which ought to characterise their learned body, the whole profession, surely, ought not to suffer. The committee also appear to have been biassed by the reports of certain keepers, and, because medicine seldom is effectual in the cure of insanity, they have too hastily drawn a conclusion, that physicians are not better qualified for superintending and directing the management and treatment of insane persons, than keepers or persons destitute of a medical education. They have also hinted at sordid conduct on the part of physicians, which, however applicable to a very few, can never attach to the profession at large; a profession which (as far as individuals are concerned, for corporations must be excepted) is exercised on the most liberal principles, and which, however, attempted to be stigmatised and cavilled at, contains in proportion to its numbers more disinterested and honourable men than any other, though, notwithstanding certain splendid exceptions, they are hardly treated and poorly remunerated.

*London, July* 14, 1814. PHILANTHROPOS.

